# Change in the incidence of Parkinson’s disease in a large UK primary care database

**DOI:** 10.1038/s41531-022-00284-0

**Published:** 2022-03-15

**Authors:** Olaitan Okunoye, Louise Marston, Kate Walters, Anette Schrag

**Affiliations:** 1 Department of Clinical and Movement Neurosciences, UCL Queen Square Institute of Neurology, University College London, London, UK; 2 Department of Primary Care and Population Health, University College London, London, UK

**Keywords:** Epidemiology, Parkinson's disease

## Abstract

Parkinson’s disease (PD) has the fastest rising prevalence of all neurodegenerative diseases worldwide. However, it is unclear whether its incidence has increased after accounting for age and changes in diagnostic patterns in the same population. We conducted a cohort study in individuals aged ≥50 years within a large UK primary care database between January 2006 and December 2016. To account for possible changes in diagnostic patterns, we calculated the incidence of PD using four case definitions with different stringency derived from the combination of PD diagnosis, symptoms, and treatment. Using the broadest case definition, the incidence rate (IR) per 100,000 person years at risk (PYAR) was 149 (95% CI 143.3–155.4) in 2006 and 144 (95% CI 136.9–150.7) in 2016. In conclusion, the incidence of PD in the UK remained stable between 2006 and 2016, when accounting for age and diagnostic patterns, suggesting no major change in underlying risk factors for PD during this time period in the UK.

## Introduction

Over the last 20 to 30 years, the prevalence of Parkinson’s disease (PD) has increased worldwide^1,2^. This is, at least partly, related to an increase in life expectancy in most countries during this time. In some studies in Western Europe and North America the incidence of PD was also reported to have increased, while the incidence of dementia and stroke is reported to have declined^1,3^. It has been postulated that an increase in risk factors for PD may underlie this increase^1,2^. However, there are few incidence studies that assess the same population at different time points with similar ascertainment methods due to difficulties in identifying people with PD in a population that is stable and generalisable over time^4^. Most studies are cross-sectional, assess prevalence, and cannot account for changes in diagnostic patterns. Amongst the few prospective incidence studies worldwide, some report an increase^4–6^, whereas others show no change^7^ or a decrease in incidence of PD over time^8–10^. Some of these studies were limited by sample size^5,9,10^ and one prospective study had a short follow-up time^4^. Furthermore, rarely has the effect of different case definitions on the incidence of PD been explored^8^.

The use of electronic medical records with appropriate case ascertainment allows for a consistent method of exploring trends in PD over time. Electronic medical records have been used in several studies for investigating trends in the incidence of conditions (for example anxiety, type 2 diabetes, lung cancer, heart failure) over time^11–17^. The issue of underreporting due to selection bias is mitigated as data are collected routinely at the time of recording a PD diagnosis in the database. A diagnosis is based on codes which are entered following letters received from the hospital specialist confirming the diagnosis. This is usually checked by the General Practitioner (GP) who flags it for data entry by administrative staff. The validity of significant diagnoses in primary care databases is high^18,19^. In a previous study, 90% of PD diagnoses (using diagnosis code and at least two prescriptions of antiparkinsonian medication) in the General Practice Research Database (GPRD), were validated as true cases when compared to paper records from a random sample of patients^20^. In addition, significant diagnoses of long-term conditions have been shown to have good specificity and sensitivity in primary care records^21,22^. However, stringent definitions of diagnosis may miss cases that can be identified using codes of symptoms and prescriptions. The limitations in terms of diagnostic accuracy can therefore at least partly be mitigated by using different case definitions of varying stringency.

In order to identify changes in incidence of PD in the UK, we therefore examined age-adjusted incidence rates of PD in a UK primary care database, using the same ascertainment methods over time, employing several definitions to account for changes in diagnostic patterns over time.

## Results

### Incidence of Parkinson’s disease

The overall crude incidence rate of PD between 2006 and 2016 as defined by the four case definitions was (1) 57 per 100,000 PYAR (95% CI: 56–58) using PD diagnosis Read codes and at least 2 prescriptions of antiparkinsonian medication; (2) 70 per 100,000 person years at risk (PYAR) (95% CI: 68–71) using solely PD diagnosis Read codes; (3) 75 per 100,000 PYAR (95% CI: 73–76) using PD diagnosis OR symptom Read codes; (4) 140 per 100,000 PYAR (95% CI: 138–141) using PD diagnosis OR symptom Read codes OR at least one prescription of antiparkinsonian medication-(broadest case definition) (Table 1 and Supplementary Tables 1 to 3).

**Table 1:** Incidence of Parkinson’s disease from 2006 to 2016 by sociodemographic factors, calendar year, and region using the broadest (most sensitive) case definition (diagnosis Read code OR symptom Read code OR at least one prescription of any antiparkinsonian medication).

Overall	Number of cases	Person-years (100,000)	Incidence of PD Rate per 100,000 (95% CI)	Adjusted ^a^IRR (95% CI)
	24,487		140.00 (138.00–141.00)	
**Age, years**				
50–54	1506	33.43	45.04 (42.82–47.38)	(Reference)
55–59	1835	30.52	60.13 (57.44–62.94)	1.34 (1.25–1.44)
60–64	2471	28.97	85.30 (82.01–88.73)	1.91 (1.79–2.05)
65–69	3234	24.83	130.27 (125.86–134.84)	2.90 (2.72–3.09)
70–74	3996	19.71	202.72 (196.53–209.11)	4.53(4.26–4.82)
75–80	4523	15.89	285.03 (276.84–293.46)	6.46 (6.08–6.86)
80–84	3781	11.60	325.90 (315.68–336.45)	7.40 (6.95–7.87)
85–89	2308	6.97	331.30 (318.05–345.09)	7.53 (7.03–8.06)
90–94	715	2.84	252.15 (234.33–271.32)	5.81 (5.29–6.38)
95+	118	0.80	147.77 (123.38–176.99)	3.29 (2.69–4.01)
**Sex**				
Male	12599	83.14	151.55 (148.92–154.22)	(Reference)
Female	11888	92.39	128.67 (126.38–131.01)	0.76 (0.74–0.78)
**Townsend quintile**				
1	6030	45.0	147.12 (141.49–152.98)	(Reference)
2	5442	39.6	144.95 (140.47–149.57)	1.07(1.02–1.12)
3	4642	34.0	137.53 (133.92–141.23)	1.03(0.99–1.07)
4	3900	26.9	136.70 (132.83–140.69)	0.99 (0.95–1.03)
5 (Most deprived)	2520	17.1	133.86 (130.52–137.28)	0.98 (0.94–1.02)
Missing	1953	12.9	151.20 (144.6–158.00)	
**Year**				
2006	2357	15.8	149.22 (143.32–155.37)	(Reference)
2007	2277	16.4	139.20 (133.60–145.04)	0.92 (0.87–0.98)
2008	2289	16.8	136.51 (131.03–142.22)	0.91 (0.86–0.97)
2009	2222	17.0	130.92 (125.58–136.47)	0.86 (0.80–0.91)
2010	2325	16.7	138.88 (133.35–144.64)	0.91 (0.86–0.97)
2011	2273	17.1	133.19 (127.82–138.78)	0.88 (0.83–0.93)
2012	2279	17.3	131.77 (126.47–137.30)	0.86 (0.81–0.92)
2013	2423	16.9	143.53 (137.93–149.46)	0.92 (0.87–0.98)
2014	2394	16.1	149.13 (143.27–155.23)	0.96 (0.91–1.02)
2015	1972	13.9	141.56 (135.45–147.95)	0.89 (0.83–0.95)
2016	1675	11.7	143.66 (136.94–150.71)	0.92 (0.86–0.99)
**Region**				
North East	420	3.6	116.77 (106.12–128.49)	(Reference)
East Midlands	501	3.6	138.40(126.79–151.06)	1.14 (0.92–1.42)
East of England	1439	9.5	151.16 (143.55–159.17)	1.25 (1.03–1.51)
London	2155	17.0	126.50 (121.27–131.96)	1.04 (0.87–1.25)
North West	2290	16.7	136.81 (131.32–142.53)	1.16 (0.97–1.39)
Northern Ireland	1235	7.2	172.18 (162.84–182.05)	1.51 (1.25–1.83)
Scotland	4010	28.7	139.58 (135.33–143.97)	1.22 (1.02 to 1.44)
South Central	2462	18.7	131.63 (126.53–136.94)	1.09 (0.91 to 1.31)
South East Coast	2618	18.5	141.86 (136.53–147.40)	1.16 (0.97 to 1.39)
South West	2157	14.5	148.56 (142.43–154.97)	1.21 (1.01 to 1.45)
Wales	2841	19.9	142.90 (137.74–148.25)	1.17 (0.98 to 1.40)
West Midlands	1899	14.2	133.60 (127.73–139.75)	1.11 (0.92 to 1.33)
Yorkshire & Humber	459	3.3	137.50 (125.48–150.67)	1.16 (0.92 to 1.45)

*PD* Parkinson’s disease, *CI*, Confidence intervals.

^a^IRR adjusted for age, gender, calendar year, social deprivation, and UK regions.

### Trends in the incidence of Parkinson’s disease over time

**Fig. 1:**
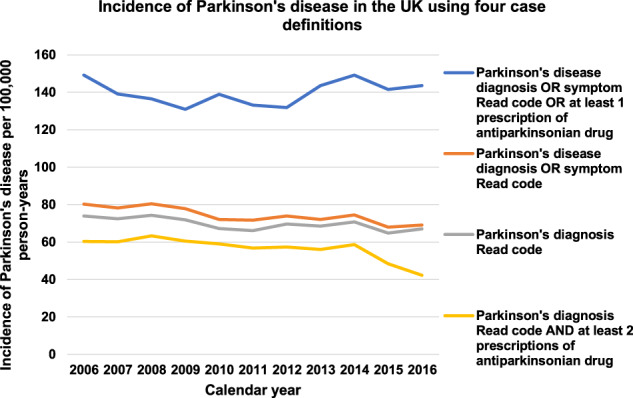
Graphs showing incidence of Parkinson’s disease in UK between 2006 and 2016 using four case definitions. The topmost graph shows the incidence using the broadest case definition. The three lower graphs show the incidence using the more stringent case definitions.

The incidence of PD using the broadest definition remained stable between 2006 and 2016 after adjusting for age, gender, calendar year, social deprivation and region with some fluctuations over this time (Fig. 1). The incidence rate of PD using this definition was 149.20 cases in 2006 and 143.70 cases per 100,000 PYAR in 2016. Using the more stringent definitions, there was a slight declining trend over time (Fig. 1, Table 1 and Supplementary Tables 1 to 3) but this was not consistently different to the baseline year.

### Relationship between incidence of Parkinson’s disease and sociodemographic factors

**Fig. 2:**
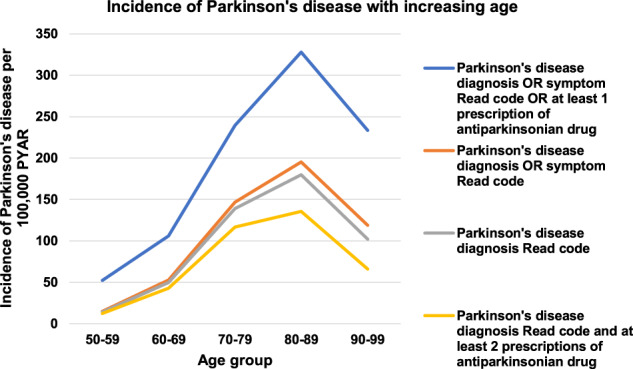
Incidence of Parkinson’s disease in THIN increasing with increasing age between 2006 and 2016 using all case definitions. The topmost graph shows the incidence using the broadest case definition. The three lower graphs show the incidence using the more stringent case definitions.

Women had a lower incidence than men for all case definitions. Using the broadest case definition, the incidence rate (IR) per 100,000 PYAR was 151.55 for men and 128.67 for women (Incidence rate ratio (IRR): 0.76 (95% CI 0.74–0.78). Overall, the incidence of PD increased with increasing age and peaked between 80 and 89 years (for all case definitions) for example, at 327.93 per 100,000 PYAR for the broadest (sensitive) case definition (Table 1 and Fig. 2 and Supplementary Tables 1 to 3).

**Fig. 3:**
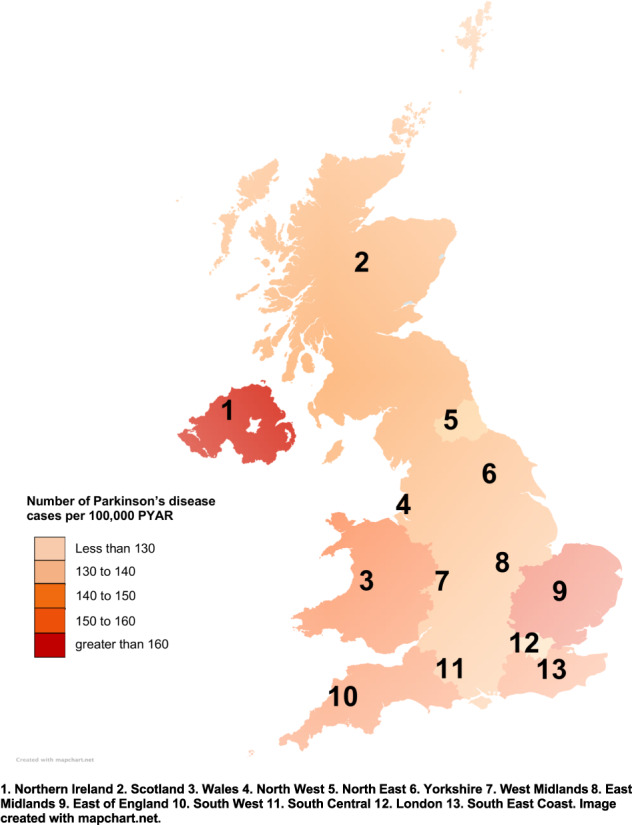
Incidence of Parkinson’s disease by regions of the UK using the broadest case definition. Incidence of Parkinson’s disease using the broadest case definition (diagnosis Read code OR symptom Read code OR at least 1 prescription of antiparkinsonian medication) per 100,000 PYAR by former Strategic Health Authority Regions from 2006 to 2016.

The highest incidence of PD was seen in Northern Ireland for all case definitions. For the broadest case definition, the incidence of PD for Northern Ireland was 172.00 per 100,000 PYAR in comparison to North East region which had the lowest incidence at 116.80 per 100,000 PYAR for this case definition (IRR: 1.51 (95% CI 1.25–1.83)). Within England, the East of England had the highest incidence of Parkinson’s disease for all case definitions (Table 1 and Fig. 3 and Supplementary Tables 1 to 3).

The incidence of PD was slightly lower in people from the most deprived areas compared to those from the least deprived areas. For the broadest case definition, the IRR was 0.98 (95% CI 0.94–1.02) with an IR of 133.86 per 100,000 PYAR in the most deprived quintile and of 147.12 in the least deprived quintile. (Table 1 and Supplementary Tables 1 to 3).

## Discussion

We found that the incidence of PD in a large UK primary care database was stable using the broadest case definition of PD (PD diagnosis OR symptom OR at least one prescription of antiparkinsonian medication) between 2006 and 2016. Using stricter case definitions, the incidence of PD decreased slightly, particularly using the strictest definition requiring a PD diagnosis Read code and at least 2 prescriptions of antiparkinsonian medication. It is likely that the decrease in incidence with the stricter case definitions reflects changes in diagnostic practice or coding by general practices^8^. The higher rate in incidence using prescription data may be more accurate as there is no incentive for PD coding in primary care and combining prescriptions of antiparkinsonian medications in addition to diagnosis codes may be a better reflection of true actual incidence rates. The Health Improvement Network (THIN) is recognized to be a suitable database for work in drug utilization due to its accuracy in prescription coding^23^. It is therefore likely that the broadest case definition is the most reliable and consistent in reflecting a diagnosis of PD in primary care.

Worldwide, the age-adjusted incidence of PD has been estimated to have increased by 6.6% between 1990 and 2019 in the Global Burden of Disease study^24,25^. Much of this change may reflect changes in the still high rates of underdiagnosis, particularly in health care systems with low availability of neurological services. Few studies directly exploring changes in incidence rates in the same population have been published, and these have provided inconsistent results. A longitudinal study in the US found an increase in age-adjusted incidence rates of PD for men, particularly over the age of 70 years, from 1976 to 2005^5^. For a more recent time period, a study from Finland reported a slight increase in age-adjusted incidence of PD between 1997 and 2014^6^. Additionally, a study in Taiwan reported a slight increase in PD incidence from 2002 to 2009 from 33.5 to 36.6 per 100,000 based on a national health service insurance database^26^, and a study from South Korea reported an increase in the period from 2010 to 2015 from 73.2 per 100,000 to 88.7 per 100,000 among people aged 50 years and more^4^. On the other hand, a previous US study in Olmsted county did not report a change in incidence of PD between 1976 and 1990^27^, and in a US study using Medicare data the incidence of PD remained stable between 1992 and 2005^7^. In a Canadian study the incidence of PD also remained relatively constant between 1990 and 2007^28^. Another study from Taiwan reported a decrease in overall incidence from 35.3 per 100,000 to 28.8 per 100,000 from 2005 to 2011^10^. The Rotterdam study from the Netherlands reported a stark decline in PD incidence from 1990 to 2010^9^. Our results in the UK do not reach back as long as some of these studies, but our own previous study conducted in the same database over an earlier time period, had similarly found a stable incidence rate using a broad definition^8^. Another study in the UK^29^, using a different but comparable dataset, also reported no change in incidence rates between 2011 and 2015.

It is unclear why there are differences in trends in incidence between these studies in different geographical regions and time periods. It is possible that there are environmental or genetic factors that differ between geographical areas and over time. For example, smoking which is known to be negatively associated with PD has become less prevalent in many countries, but so have exposure to pesticide and other environmental risk factors that have, conversely, been associated with an increased risk of PD^30^. However, it is also possible that despite best efforts methodological differences, such as residual changes in diagnostic coding or case ascertainment may account for discrepancies in these studies^31^, and greater awareness and higher diagnostic rates are likely to be particularly relevant where a decrease in incidence in the older age groups are seen^4^, as difficulty with movement may be misinterpreted as being due to comorbidities.

Overall, the incidence rates of PD in this study are comparable to other published studies in European populations^32,33^. In addition, a recent study by Parkinson’s UK using a different but comparable dataset^29^, reported an incidence rate of 71 per 100,000 in individuals aged 50–94 years between 2011 and 2015 using the diagnostic code for PD, which is similar to our incidence rate of 70 per 100,000 using the same diagnostic code in this age group. In keeping with other studies, we also found an increase with age^8,34–39^, except in the oldest age group (90 years and more) where the incidence rate was lower and gradually decreased. This has been reported to be due to underdiagnosis of PD in the older age groups^39,40^ due to multiple health challenges that make isolation of PD symptoms particularly difficult in this age group^40^. Men were more likely to be recorded to have a diagnosis of PD compared to women, in keeping with previous research^36,37,39,41^. The incidence of PD was highest in those who live in more affluent areas compared to those who lived in the most deprived areas. This difference was similar for all case definitions and also reported in the previous study^8^. This could reflect lower rates of health-seeking behaviour or diagnosis in lower socioeconomic groups, or could be due to confounding factors such as smoking which is linked to lower risk of PD^42,43^ and is also well established to be associated with deprivation^44,45^.

After controlling for age, gender, calendar year, region, and social deprivation, the incidence rate of PD was highest in Northern Ireland. There are no previous studies to compare but in the recent study on prevalence and incidence of PD by Parkinson’s UK incidence of PD was highest in England using another routine data source (the Clinical Practice Research Datalink (CPRD))^29^. However, the incidence rates in different areas are less robust because of smaller sample sizes than in the overall study.

The strength of this study is that the data were derived from routinely collected health records of a large population of patients from many general practices over an eleven-year time period. This allowed us to follow up a large cohort of patients, which were largely representative of the UK general population, without any major change in ascertainment method^23^. The large number of individuals included in the analysis enabled us to calculate estimates by age group, gender, socioeconomic status, calendar year, and region. In addition, the use of routinely collected prospective data captures cases without recall or selection bias in diagnosing PD in primary care. In addition, we used a definition for PD to allow for changes in diagnostic and coding patterns, as well as more stringent diagnostic definitions. All these did not suggest an increase in the incidence of PD in the UK.

Another strength of this study is that data on age, gender, prescriptions, region were complete, and the only missing data were on social deprivation. However, the incidence rates of PD were higher in those with missing data in these variables and so likely not missing at random. There is a possibility that those with missing data on social deprivation are in more affluent areas.

The main limitation of this study is that we could not confirm the diagnosis of PD and depended on clinicians recording of the diagnosis of PD in electronic medical records instead of systematic evaluation of cases. Although we used four different case definitions which involved not only diagnosis codes but treatment and symptom variables, there may still be some misclassification if a diagnosis of PD was not considered. However, a previous validation study has shown that the strictest case definition has good specificity for PD^20^.

In addition, there may be other confounding factors (such as ethnicity) which we have not accounted for due to large number of missing data. Finally, the use of GP records for investigating the incidence of PD meant that the results of the analysis are confined to those registered with a general practitioner and rates may be different in the small number of people not registered with a GP, but the numbers of the population not registered with primary care in the UK is very small (2%)^45–48^.

In conclusion, trends in recordings of routine diagnoses of PD between 2006 and 2016 did not indicate an increase of age-adjusted incidence rates of PD over this time period. This suggests that it is unlikely that there have been major changes in risk factors such as environmental toxins associated with PD in the UK during this time. Male sex, older age group, and living in the more affluent areas were the key factors associated with having PD, confirming previous studies.

## Methods

### Data source

We used electronic primary healthcare data from the IQVIA Medical Research Data (IMRD) that incorporates data supplied by The Health Improvement Network (THIN), a propriety database of Cegadim SA. This is one of the largest databases containing anonymized electronic medical records generated from more than 700 general practices and about 12 million patients’ data from all over the UK^49^ (3.7 million active patients) equivalent to 75.6 million patient years of data, covering 6.2% of UK population. All data are de-identified, processed, and validated by CSD Medical Research UK^49,50^.

THIN has data on patient demographics, disease diagnoses, symptoms, prescribed medications^23,51^, Townsend quintiles as a measure of social deprivation^52^, referrals to secondary care, and free text information. Symptoms and diagnoses are entered using the Read code classification system, a hierarchical coding system^53–55^. It is estimated that about 98% of the population of UK are registered with a General Practice (GP)^56^ and more than 90% of NHS contacts are in general practice^57^. The data quality has also been demonstrated to be high in independent validation studies^18,58^.

### Study population and time period

General practices that contributed data to THIN between January 2006 and December 2016 were used in this study. The quality of data included was assessed using two quality filters. First, is the acceptable computer usage (ACU) dates which is used to determine when a general practice was using electronic recording fully^59^ and second, is the acceptable mortality recording (AMR) date. AMR date is a measure of the quality of death records in THIN. It is the year from which an individual general practice is considered to have mortality records, which are proportional to that from the Office for National Statistics (ONS)^60^. Practices were included after the latest of the ACU and AMR dates.

All individuals aged 50 years and over that were registered with a general practice contributing data between January 1^st^, 2006 and December 31^st^, 2016 were included in the analysis.

### Identification of Parkinson’s disease cases in The Health improvement Network- (THIN)

Four case definitions with varying levels of stringency were developed to identify people with PD: (1) A PD diagnosis Read code plus at least 2 antiparkinsonian drug prescriptions. This method of identification of people with PD is the strictest (most specific) and has been validated in General Practice Research Database (GPRD), another primary health care database^20^ and used in a previous study^61^. (2) a PD diagnosis Read code alone; (3) a PD diagnosis Read code OR Read code for parkinsonian symptom, secondary and unspecified parkinsonism (excluding drug-induced parkinsonism); (4) a PD diagnosis Read code OR symptom Read code OR at least one antiparkinsonian drug prescription from 5 classes of antiparkinsonian medication: Levodopa-containing medications, Dopamine-receptor agonists, Amantadine, Monoamine-oxidase--B inhibitors-rasagiline and selegiline and Catechol-O-methyl transferase inhibitors (entacapone and tolcapone). This is the broadest and most sensitive case definition. Read code lists for diagnosis and symptoms of Parkinson’s disease and drug code list for antiparkinsonian medications were identified using developed methods^54^ (Supplementary Figs. 1 and 2).

The earliest record of the PD diagnosis Read code, symptom, or drug code for antiparkinsonian drug prescription were considered as the index date. In order to distinguish incident and prevalent cases, the first diagnosis or symptom or prescription date had to be at least six months following the patient’s registration with a GP practice^58^. Thus, we excluded all individuals with PD diagnosis in the first six months after registration with a practice as this may represent retrospective recording rather than a true new recording of PD^58^. We also excluded those with restless leg syndrome without PD who might have been exposed to treatment with dopamine agonists.

Patients entered the cohort on the latest of: the start date of study period (January 1st 2006), acceptable mortality reporting (AMR) date, acceptable computer usage (ACU) date, 50th birthday or GP registration plus six months for our analysis on the incidence of PD. Patients exited the cohort on the earliest date of PD diagnosis, left the GP practice, died, last data recorded in THIN, or the study period ended (December 31st 2016).

### Statistical analysis

The overall crude incidence of PD recording using all four case definitions was estimated as the number of cases per 100,000 Person Years At Risk (PYAR).

This incidence of PD was calculated by adding the total number of patients with a first recording of diagnosis or symptom or prescription plus six months, between 2006 and 2016 and this number was then divided by the total person years of follow-up for all patient records for this time period.

The crude incidence rates of PD recording using all four case definitions were estimated by age group, gender, social deprivation, calendar year, and region, restricting the person years of follow-up according to the category in question. For descriptive analysis, the age group was defined by 5-year intervals: 50–54, 55–59, 60–64, 65–69, 70–74, 75–79, 80–84, 85–89, 90–94, and 95 years and over. Gender was defined as male and female. Townsend quintile was used to assess the level of social deprivation. The score ranges from 1 to 5, with 1 being the most affluent and five indicating the highest level of deprivation. The UK regions were based on the former Strategic Health Authorities. These were: East Midlands, East of England, London, North-East, North-West, South Central, South East Coast, South West, West Midlands, Yorkshire and Humber (all in England), Northern Ireland, Scotland and Wales.

Multivariable Poisson regression analysis was conducted to investigate the incidence (using the four case definitions) by age group, gender, Townsend quintile, calendar year, and region, adjusting for the respective variables included in this model. In order to fit the Poisson model to generate a rate ratio, the coefficients were exponentiated with person-time specified as the exposure.

Using all four case definitions, annual incidence rates were calculated in order to explore trends in the incidence of Parkinson’s disease recordings over time.

Additional exploratory work was conducted by calculating and comparing incidence rates of PD at a similar time period (2011–2015) to Parkinson’s UK report using similar diagnosis Read codes: F12..00 Parkinson’s disease, F120.00 Paralysis agitans and F12z.00 Parkinson’s disease not otherwise specified) (Supplementary Table 4). Stata (version 16MP) was used to carry out all statistical analyses^62^.

### Ethics

In 2003, the NHS South-east Multi-Centre Research Ethics Committee gave approval for the use of THIN overall. This study was approved by IQVIA Medical Research’s Scientific Review Committee in June 2019. (SRC Reference Number: 19THIN034).

### Reporting Summary

Further information on research design is available in the Nature Research Reporting Summary linked to this article.

## Supplementary information

Supplementary materials

Reporting summary

## Data Availability

The authors have obtained the data for this study from IQVIA through a research license and do not own the dataset used and do not have permission to share the data. Access to THIN can be obtained through IQVIA by applying for a research license. More information on the availability of THIN data is available in the following URL https://www.iqvia.com/locations/uk-and-ireland/thin and permissions for data access can be obtained through https://www.iqvia.com/contact/general. The authors accessed the data in the same manner and had no special privileges to the data.
