# Supplementary material for: Change in the incidence of Parkinson’s disease in a large UK primary care database
Source: NPJ Parkinsons Dis. 2022 Mar 15;8:23. doi: 10.1038/s41531-022-00284-0 (PMC8924194; doi:10.1038/s41531-022-00284-0)
Supplement: Supplementary file 1 — Supplementary materials [file 41531_2022_284_MOESM1_ESM.pdf]

Supplementary materials

Supplemental Figure 1: Read codes used for case identification.

PD diagnosis Read codes

Read code	Description	Type
Eu02300	[X]Dementia in Parkinson's disease	diagnosis
F11x900	Cerebral degeneration in Parkinson's disease	diagnosis
F12..00	Parkinson's disease	diagnosis
F12z.00	Parkinson's disease NOS	diagnosis
F130300	Parkinsonism with orthostatic hypotension	diagnosis

PD diagnosis Read codes and symptom Read codes

Read code	Description	Type
2944.00	O/E - muscle rigid - cogwheel	symptom
2944.11	O/E - cog wheel rigidity	symptom
297A.00	O/E - Parkinsonian tremor	symptom
2987.00	O/E -Parkinson flexion posture	symptom
2987.11	O/E - Parkinson posture	symptom
2994.00	O/E-festination-Parkinson gait	symptom
2994.11	O/E - Parkinson gait	symptom
A94y100	Syphilitic parkinsonism	diagnosis
Eu02300	[X]Dementia in Parkinson's disease	diagnosis
F11x900	Cerebral degeneration in Parkinson's disease	diagnosis
F12..00	Parkinson's disease	diagnosis
F123.00	Postencephalitic parkinsonism	diagnosis
F12X.00	Secondary parkinsonism, unspecified	diagnosis
F12z.00	Parkinson's disease NOS	diagnosis
F130300	Parkinsonism with orthostatic hypotension	diagnosis
F13z300	Akinetic rigid syndrome	diagnosis
Fyu2100	[X]Other secondary parkinsonism	diagnosis
Fyu2200	[X]Parkinsonism in diseases classified elsewhere	diagnosis
Fyu2900	[X]Secondary parkinsonism, unspecified	diagnosis

5 classes of antiparkinsonian medications:
Levodopa-containing medications
Dopamine-receptor agonists
Amantadine
Monoamine oxidase-B inhibitors-MAOB-I (rasagiline and selegiline hydrochloride)
Catechol-O-methyl transferase inhibitors-COMT-I (entacapone and tolcapone)

Supplemental Figure 2: Four case definitions using PD diagnosis Read codes, Symptom Read codes and Treatment codes (drug codes)

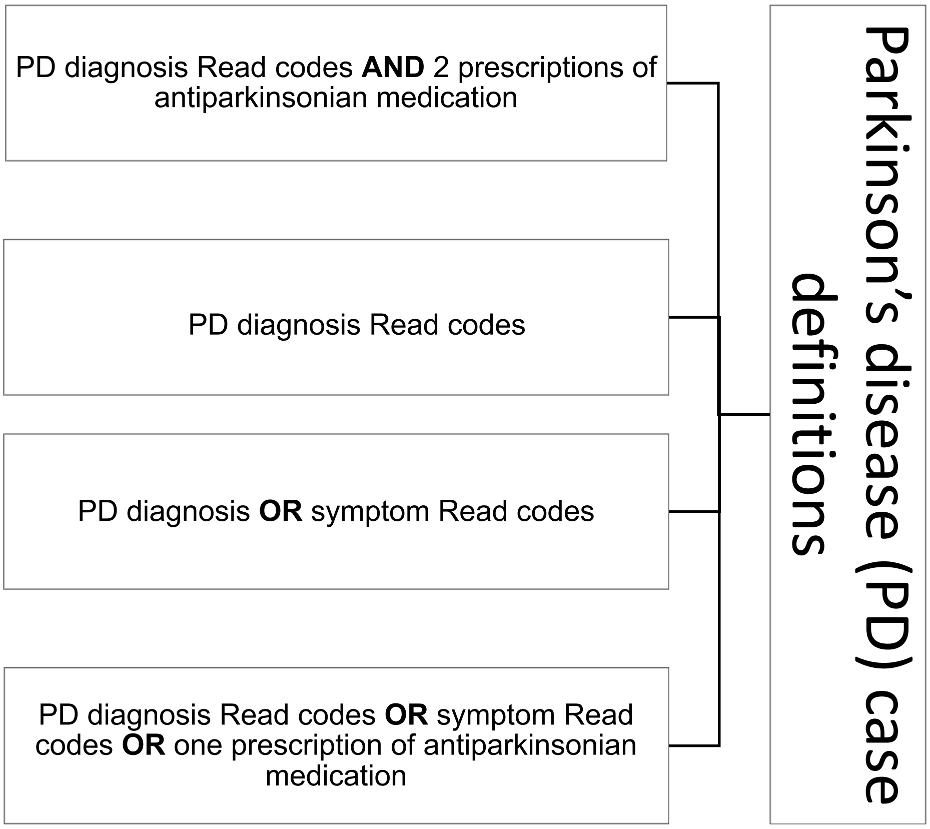

Supplemental Table 1: Incidence of Parkinson's disease between 2006 and 2016 by age, gender, social deprivation, calendar year and region using the strictest (most specific) case definition (diagnosis Read codes AND at least 2 prescriptions of any antiparkinsonian medication).

	Number of cases	Person-years (100,000)	Incidence of PD Rate per 100,000 (95% CI)	Adjusted *IRR (95% CI)
**Overall**	10104		57 (56-58)	
**Age, years**				
50-54	294	33.60	8.75 (7.80-9.81)	(Reference)
55-59	497	30.68	16.20 (14.84-17.69)	1.86 (1.60-2.16)
60-64	892	29.15	30.60 (28.66-32.68)	3.57 (3.11-4.09)
65-69	1435	25.02	57.36 (54.47-60.40)	6.65 (5.84-7.58)
70-74	1971	19.90	99.05 (94.77-103.52)	11.62 (10.22-13.20)
75-80	2220	16.05	138.36 (132.72-144.24)	16.46 (14.50-18.69)
80-84	1678	11.75	142.85 (136.17-149.85)	17.26 (15.17-19.65)
85-89	874	7.06	123.78 (115.84-132.27)	15.30 (13.33-17.57)
90-94	214	2.88	74.42 (65.09-85.09)	9.77 (8.14-11.73)
95+	29	0.81	35.88 (24.93-51.63)	4.65 (3.11-6.96)
**Sex**				
Male	6135	83.83	73.18 (71.34-75.04)	(Reference)
Female	3969	93.05	42.66 (41.35-44.00)	0.52 (0.50-0.55)
**Townsend quintile**				
1	2811	45.36	61.97 (59.72-64.30)	(Reference)
2	2320	39.87	58.19 (55.87-60.61)	0.91 (0.86-0.97)
3	1901	34.22	55.55 (53.11-58.10)	0.88 (0.82-0.93)
4	1441	27.13	53.12 (50.45-55.93)	0.83 (0.78-0.89)
5 (Most deprived)	895	17.28	51.80 (48.52-55.30)	0.82 (0.76-0.89)
Missing	736	13.02	56.53 (52.59-60.76)	
**Year**				
2006	961	15.91	60.42 (56.72-64.36)	(Reference)
2007	990	16.47	60.12 (56.48-63.97)	0.98 (0.90-1.08)
2008	1070	16.89	63.36 (59.68-67.27)	1.05 (0.96-1.14)
2009	1035	17.10	60.54 (56.97-64.35)	0.97 (0.88-1.06)
2010	994	16.87	58.93 (55.38-62.72)	0.94 (0.86-1.03)
2011	975	17.20	56.70 (53.25-60.37)	0.91 (0.83-1.00)
2012	999	17.43	57.32 (53.87-60.98)	0.93 (0.85-1.02)
2013	953	17.02	56.00 (52.55-59.67)	0.90 (0.82-0.98)
2014	949	16.19	58.62 (55.00-62.47)	0.93 (0.85-1.02)
2015	681	14.05	48.46 (44.95-52.23)	0.76 (0.69-0.84)
2016	497	11.77	42.24 (38.69-46.12)	0.67 (0.60-0.75)
**Region**				
Wales	1002	20.03	50.02 (47.02-53.22)	(Reference)
East Midlands	197	3.65	53.99 (46.95-62.08)	0.98 (0.82-1.19)
East of England	623	9.60	64.90 (60.00-70.20)	1.22 (1.07-1.39)
London	1002	17.16	58.40 (54.90-62.13)	1.21 (1.08-1.35)
North East	212	3.62	58.60 (51.22-67.05)	1.17 (0.97-1.41)
North West	918	16.88	54.37 (50.97-58.01)	1.07 (0.95-1.20)
Northern Ireland	500	7.25	69.00 (63.21-75.32)	1.46 (1.28-1.68)
Scotland	1526	28.95	52.71 (50.13-55.43)	1.15 (1.04-1.27)
South Central	1121	18.84	59.50 (56.12-63.01)	1.13 (1.01-1.26)
South East Coast	1194	18.58	64.25 (60.71-68.00)	1.24 (1.11-1.38)
South West	871	14.64	59.50 (55.67-63.58)	1.09 (0.97-1.22)
West Midlands	761	14.32	53.13 (49.49-57.04)	1.01 (0.89-1.14)
Yorkshire & Humber	177	3.36	52.65 (45.44-61.01)	1.02 (0.84-1.25)

*IRR adjusted for age, gender, year, Townsend quintile and region.

Supplemental Table 2: Incidence of Parkinson's disease between 2006 and 2016 by age, gender, social deprivation calendar year and region using diagnosis Read codes.

	Number of cases	Person-years (100,000)	Incidence of PD Rate per 100,000 (95% CI)	Adjusted *IRR (95% CI)
**Overall**	12336		70 (95% CI 69-71)	
**Age band, years**				
50-54	330	33.60	9.82 (8.81-10.94)	(Reference)
55-59	573	30.68	18.67 (17.21-20.27)	1.89 (1.64-2.17)
60-64	1043	29.14	35.79 (33.68-38.01)	3.69 (3.24-4.19)
65-69	1653	25.01	66.09 (63.98-69.35)	6.77 (5.98-7.65)
70-74	2286	19.89	114.92 (110.31-119.73)	11.94 (10.59-13.46)
75-80	2704	16.03	168.64 (162.41-175.12)	17.77 (15.78-20.01)
80-84	2158	11.73	183.95 (176.35-191.88)	19.74 (17.49-22.27)
85-89	1214	7.05	172.39 (162.97-182.36)	18.97 (16.70-21.53)
90-94	334	2.87	116.42 (104.58-129.60)	13.24 (11.30-15.52)
95+	41	0.81	50.83 (37.42-69.03)	5.85 (4.16-8.24)
**Sex**				
Male	7423	83.80	88.58 (86.59-90.62)	(Reference)
Female	4913	93.01	52.83 (51.38-54.33)	0.53 (0.51-0.55)
**Townsend quintile**				
1	3349	83.80	73.84 (71.38-76.38)	(Reference)
2	2837	93.01	71.19 (68.62-73.85)	0.93 (0.89-0.98)
3	2309	83.80	67.52 (64.82-70.33)	0.89 (0.84-0.94)
4	1801	93.01	66.42 (63.4-69.56)	0.87 (0.82-0.92)
5 (Most deprived)	1127	83.80	65.26 (61.56-69.19)	0.86 (0.81-0.93)
Missing	913	93.01	70.15 (65.75-74.85)	
**Year**				
2006	1174	15.90	73.90 (69.79-78.24)	(Reference)
2007	1193	16.46	72.46 (68.46-76.69)	0.97 (0.90-1.06)
2008	1253	16.88	74.22 (70.23-78.45)	1.00 (0.92-1.09)
2009	1227	17.09	71.80 (67.89-75.93)	0.94 (0.87-1.02)
2010	1132	16.86	67.14 (63.34-71.17)	0.88 (0.81-0.96)
2011	1137	17.19	66.14 (62.41-70.10)	0.88 (0.81-0.95)
2012	1212	17.42	69.56 (65.75-73.59)	0.91 (0.84-0.99)
2013	1164	17.01	68.42 (64.60-72.47)	0.89 (0.82-0.97)
2014	1146	16.18	70.81 (66.83-75.03)	0.93 (0.85-1.01)
2015	910	14.05	64.77 (60.70-69.12)	0.84 (0.77-0.92)
2016	788	11.75	67.00 (62.49-71.85)	0.85 (0.77-0.94)
**Region**				
Wales	1225	20.02	61.20 (57.80-64.70)	(Reference)
East Midlands	249	3.64	68.30 (60.30-77.30)	1.08 (0.91-1.28)
East of England	755	9.60	78.70(73.30-84.50)	1.25 (1.11-1.42)
London	1225	17.15	71.40 (67.50-75.60)	1.23 (1.11-1.36)
North East	258	3.63	71.40 (63.20-80.60)	1.19 (1.00-1.42)
North West	1124	16.88	66.60 (62.80-70.60)	1.10 (0.99-1.23)
Northern Ireland	587	7.24	81.00 (74.70-87.90)	1.42 (1.25-1.61)
Scotland	1893	28.94	65.40 (62.50-68.40)	1.16 (1.06-1.27)
South Central	1373	18.83	72.90 (69.20-76.90)	1.16 (1.04-1.28)
South East Coast	1414	18.58	76.10 (72.20-80.20)	1.22 (1.10-1.35)
South West	1069	14.63	73.00 (68.80-77.60)	1.12 (1.01-1.25)
West Midlands	936	14.2	65.40 (61.30-69.70)	1.05 (0.94-1.18)
Yorkshire & Humber	228	3.3	67.90 (59.60-77.30)	1.12 (0.94-1.35)

*IRR adjusted for age, gender, year, Townsend quintile and region.

Supplemental Table 3: Incidence of Parkinson's disease between 2006 and 2016 by age, gender, social deprivation, calendar year and region using diagnosis Read codes OR symptom Read codes.

	Number of cases	Person-years (100,000)	Incidence of PD Rate per 100,000 (95% CI)	Adjusted *IRR (95% CI)
**Overall**	13173		75(73-76)	
**Age, years**				
50-54	353	33.60	10.51 (9.47-11.66)	(Reference)
55-59	604	30.68	19.69 (18.18-21.32)	1.87 (1.63-2.14)
60-64	1101	29.14	37.79 (35.62-40.09)	3.65 (3.23-4.14)
65-69	1755	25.0	70.19 (66.98-73.55)	6.78 (6.03-7.65)
70-74	2417	19.88	121.57 (116.82-126.52)	11.88 (10.58-13.35)
75-79	2853	16.02	178.07 (171.65-184.73)	17.64 (15.72-19.80)
80-84	2336	11.72	199.34 (191.42-207.59)	20.17 (17.95-22.67)
85-89	1327	7.04	188.54 (178.66-198.96)	19.47 (17.23-22.02)
90-94	375	2.86	130.90 (118.30-144.84)	14.13 (12.14-16.44)
95+	52	0.81	64.52 (49.17-84.67)	7.01 (5.16-9.52)
**Sex**				
Male	7908	83.76	94.41 (92.35-96.51)	(Reference)
Female	5265	92.98	56.63 (55.12-58.18)	0.53 (0.51-0.55)
**Townsend quintile**				
1	3548	45.34	78.25 (75.72-80.87)	(Reference)
2	2994	39.84	75.16 (72.51-77.90)	0.93 (0.88-0.98)
3	2473	34.19	72.32 (69.53-75.22)	0.90 (0.85-0.94)
4	1949	27.10	71.91 (68.79-75.18)	0.88 (0.83-0.94)
5 (Most deprived)	1232	17.26	71.39 (67.51-75.49)	0.89 (0.83-0.95)
Missing	977	13.01	75.09 (70.53-79.95)	
**Year**				
2006	1274	15.89	80.16 (75.87-84.68)	(Reference)
2007	1286	16.46	78.14 (73.99-82.53)	0.97 (0.89-1.05)
2008	1357	16.87	80.42 (76.26-84.82)	1.00 (0.93-1.09)
2009	1329	17.08	77.81 (73.73-82.10)	0.94 (0.87-1.02)
2010	1214	16.85	72.09 (68.1-76.20)	0.88 (0.81-0.95)
2011	1231	17.18	71.64 (67.75-75.75)	0.88 (0.81-0.95)
2012	1286	17.42	73.84 (69.91-77.98)	0.90 (0.83-0.97)
2013	1226	17.01	72.09 (68.17-76.24)	0.86 (0.80-0.94)
2014	1205	16.18	74.49 (70.39-78.81)	0.90 (0.83-0.98)
2015	954	14.04	67.93 (63.79-72.38)	0.81 (0.74-0.89)
2016	811	11.76	68.98 (64.39-73.89)	0.81 (0.74-0.89)
**Region**				
Wales	1305	20.0	65.20 (61.76-68.83)	(Reference)
East Midlands	271	3.60	74.33 (65.99-83.73)	1.09 (0.92-1.29)
East of England	820	10.0	85.50 (79.85-91.56)	1.27 (1.13-1.43)
London	1310	17.1	76.43 (72.40-80.68)	1.23 (1.11-1.36)
North East	275	3.60	76.09 (67.60-85.63)	1.19 (1.00-1.42)
North West	1216	16.9	72.08 (68.14-76.25)	1.12 (0.99-1.24)
Northern Ireland	612	7.20	84.64 (78.20-91.61)	1.39 (1.22-1.58)
Scotland	2021	28.9	69.86 (66.88-72.98)	1.16 (1.06-1.27)
South Central	1475	18.8	78.34 (74.46-82.46)	1.17 (1.05-1.29)
South East Coast	1488	18.6	80.12 (76.15-84.30)	1.20 (1.08-1.33)
South West	1149	14.6	78.55 (74.13-83.22)	1.13 (1.02-1.26)
West Midlands	980	14.3	68.48 (64.32-72.89)	1.04 (0.93-1.16)
Yorkshire & Humber	250	3.40	74.45 (65.76-84.27)	1.14 (0.95-1.36)

*IRR adjusted for age, gender, year, Townsend quintile and region

Supplemental Table 4: Comparing incidence rates (per 100,000 PYAR) of Parkinson's disease by age-group (2011-2015) with results of the Parkinson's UK study using similar case definitions (diagnostic Read codes).

THIN

Gender	Age	N	Person years	Incidence Rate per 100,000	95% CI lower	95% CI Upper
All	50-54	335	33.9	9.9	8.9	11.0
All	55-59	581	30.9	18.8	17.3	20.3
All	60-64	1057	29.4	35.9	33.8	38.2
All	65-69	1662	25.3	65.7	62.7	69.0
All	70-74	2303	20.1	114.6	110.0	119.3
All	75-79	2731	16.2	168.6	162.4	175.1
All	80-84	2170	11.8	183.2	175.6	191.1
All	85-89	1228	7.1	172.7	163.3	182.6
All	90-94	337	2.9	116.5	104.7	129.6
All	95-99	41	0.8	50.4	37.1	68.5

CI Confidence Interval

CPRD

Gender	Age	N	Person years	Incidence Rate per 100,000	95% CI lower	95% CI Upper
All	50-54	134	15.8	9.1	7.6	10.7
All	55-59	232	14.8	18.2	15.9	20.7
All	60-64	398	11.9	33.5	30.3	37.0
All	65-69	714	11.5	62.3	57.8	67.1
All	70-74	969	8.5	113.4	106.3	120.7
All	75-79	1207	7.1	173.5	163.9	183.6
All	80-84	1032	5.3	195.5	183.7	207.8
All	85-89	615	3.3	186.3	171.9	207.1
All	90-94	179	1.5	115.1	98.9	133.3
All	95-99	NA	NA	NA	NA	NA
